# Angry, Scared, and Unsure: Mental Health Consequences of Contaminated Water in Flint, Michigan

**DOI:** 10.1007/s11524-016-0089-y

**Published:** 2016-11-02

**Authors:** Courtney A. Cuthbertson, Cathy Newkirk, Joan Ilardo, Scott Loveridge, Mark Skidmore

**Affiliations:** Michigan State University, East Lansing, MI USA

**Keywords:** Water, Mental health, Substance use, Infrastructure management, Behavioral health, Flint

## Abstract

Natural and manmade crises impact community-level behavioral health, including mental health and substance use. This article shares findings from a larger project about community behavioral health, relevant to the ongoing water crisis in Flint, Michigan, using data from a larger study, involving monthly surveys of a panel of key informants from Genesee County. The data come from open-response questions and are analyzed as qualitative data using grounded theory techniques. Although respondents were not asked about the water issues in Flint, participants commented that the water situation was increasing stress, anxiety, and depression among the city’s population. Participants thought these mental health issues would affect the entire community but would be worse among low-income, African American populations in the city. Mental health consequences were related not only to the water contamination but to distrust of public officials who are expected and have the authority to resolve the issues. The mental health effects of this public health crisis are significant and have received inadequate attention in the literature. Public health response to situations similar to the water issues in Flint should include sustained attention mental health.

## Introduction

The built environment can affect mental health directly through factors such as housing quality and indirectly through factors such as a sense of personal control over one’s surroundings.^1^ For example, toxic waste landfills are more likely to be sited near communities of color.^2^ People of low socioeconomic status and communities of color are more likely to be exposed to hazardous waste, with similar findings with regard to air and water pollution.^3^ Living in close proximity to industrial activity negatively impacts mental health, with disproportionately higher impacts on people of color.^4^

People who experience shocks from natural or manmade disasters have higher rates of mental illness, including major depression and posttraumatic stress disorder.^5^^–^ 7 Neighborhoods with higher collective efficacy generally have better reported overall health^8^ and better mental health outcomes in the face of natural disasters.^9^^,^ 10

While clinically diagnosable mental illnesses may be triggered or influenced by stressful events such as disasters and environmental injustices, we must point out that this differs from the psychological distress individuals may experience in similar or identical situations. Distress is often viewed as a natural reaction to stressful events or life conditions,^11^ as a psychosocial response to environmental disasters, based on direct and perceived individual impact and risk to health.^12^ Distress and disorder should be considered with relation to the experience of symptoms (severity, duration) relative to what one may expect based on the stressfulness of a particular situation,^11^ such that distress occurs in situations where experienced symptoms are less than or equal to what is expected based on stressful events or circumstances, and disorders occur when experienced symptoms are greater than what would be expected. Experiences and interpretations of distress vary by social positionality.

In April 2014, the City of Flint began treating its own drinking water from the Flint River. Prior to that time, the city received treated water from the Detroit Water and Sewer Department (DWSD). Soon after the switch in water source, residents voiced their concerns that the water was discolored, had a bad smell and taste, and was causing health problems. State and city officials initially dismissed many of the complaints as insignificant. Even when the water source was switched back to DWSD in October 2015 after state officials publicly acknowledged problems with the water, some of those problems persisted. News accounts of citizens’ reactions to the situation note distrust of government because of the discounting of residents’ concerns about water and the length of time it took to act. Residents’ reactions include anger, disillusionment, abandonment, and feeling as though their government officials cared little about them. Some physical health impacts have been described by residents and researchers, such as elevated blood lead levels,^13^ but the impact of the mental stress associated with the water crisis received substantially less attention to date. The media have compared the water situation in Flint to New Orleans after Hurricane Katrina, in terms of the disasters’ human health costs, the disproportionate impact on black and low-income residents, and the seemingly slow governmental response.^14^

Flint has long been a site of environmental injustice.^15^ Contemporary Flint is often characterized as a disadvantaged community, with a median income of just over $24,000,^16^ which is less than half the national average. In disadvantaged communities, social capital may mediate the negative effects of low income or income inequality on health status.^17^^,^ 18 However, people with low social capital are more likely to experience mental illnesses.^19^ In Flint specifically, neighborhood social capital has been directly, negatively related to stress and depressive symptoms.^20^ The purpose of the current article is to investigate the mental health impacts of the problems with Flint’s water, as described by community leaders in Genesee County.

## Methods

The aim of the project was to create community monitoring or sentinel surveillance systems for mental health and substance use issues, piloting three methods across 17 different communities around the USA. The project was approved by the Institutional Review Board at Michigan State University. Partnering communities were selected through an open call for proposals and selected based on demonstrated capacity to successfully complete the project, as well as demographic and geographic diversity. For purposes of the current paper, only the results from the Genesee County, Michigan, partnering community, which encompasses the city of Flint, are considered.

**FIG. 1:**
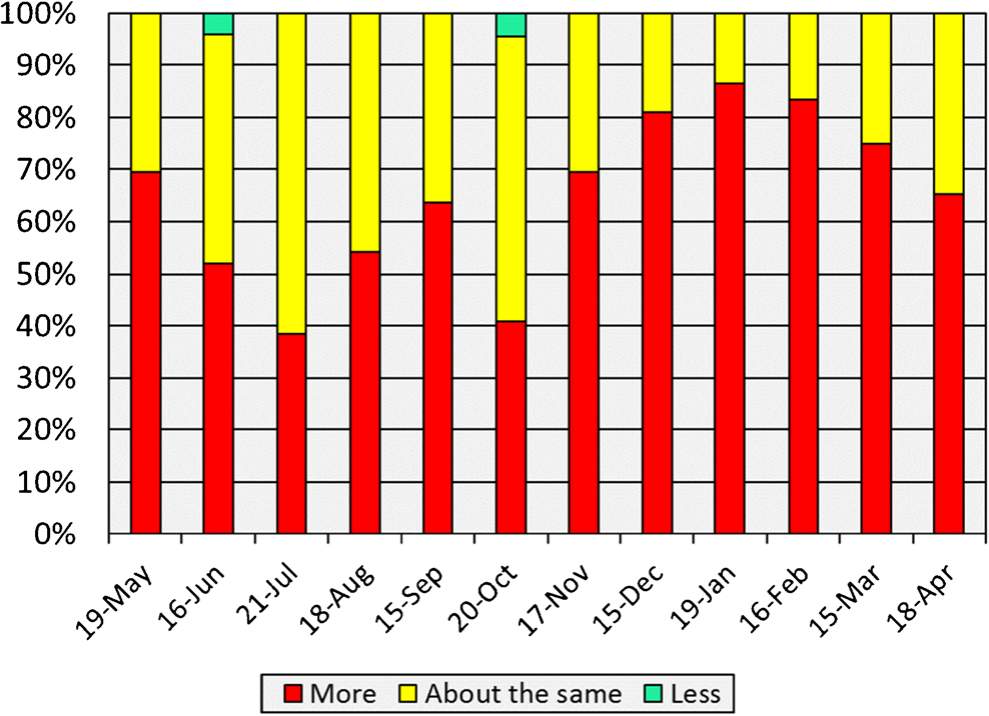
Participants’ perceptions of changes in stress in Genesee County.

Genesee County was part of a group of communities testing community monitoring via monthly surveys of a panel of 30 key informants. Panelist expertise included health services, substance use prevention, health-related non-governmental organizations, disability service organizations, schools, and researchers. Panelists were asked to complete a survey about changes in 30 behavioral health-related issues, including specific mental health and substance use disorders, each month for 1 year. The survey, developed by the project’s technical committee, was administered online via Qualtrics. Participants had 1 week to complete each monthly survey, with reminders sent in the middle of the survey period to any participant who had not yet responded. The survey asks panelists to identify any new behavioral health issues that were not present in the community before, as well as whether any community events occurred that may have impacted behavioral health. Responses to these open questions were not required for survey completion. Participants received $10 per survey completed. As shown in Fig. 1, panelists perceived stress to become much worse as the water crisis unfolded during the course of the surveys; panelists perceived a brief reduction of stress around the time of the switch back to DWSD, but that it rebounded as the long-term effects of lead exposure on individuals, families, and infrastructure became more widely known and as media attention increased.

Data for this paper come from 12 monthly surveys completed by panelists in Genesee County. More specifically, the data come from the open-response questions about what new issues and community events emerged since the previous survey, as well as open-response comment fields where respondents could mention issues related to a subset of behavioral health problems. The data were analyzed as qualitative comments using grounded theory, including open and focused coding, and concept building techniques such as memoing.^21^^–^ 23 Within grounded theory, researchers start with the data and build theory from empirical findings to compare with existing knowledge. Each open-response comment represented one data point for coding. The open-response comment fields were coded using open coding to determine the most common themes from the data and again using focused coding to refine themes and develop more detailed definitions. Concurrently with coding, memoing aided in drawing connections between themes. All authors reviewed the data, one author completed coding and memoing, and all authors reviewed themes and details for accuracy.

## Results

**FIG. 2:**
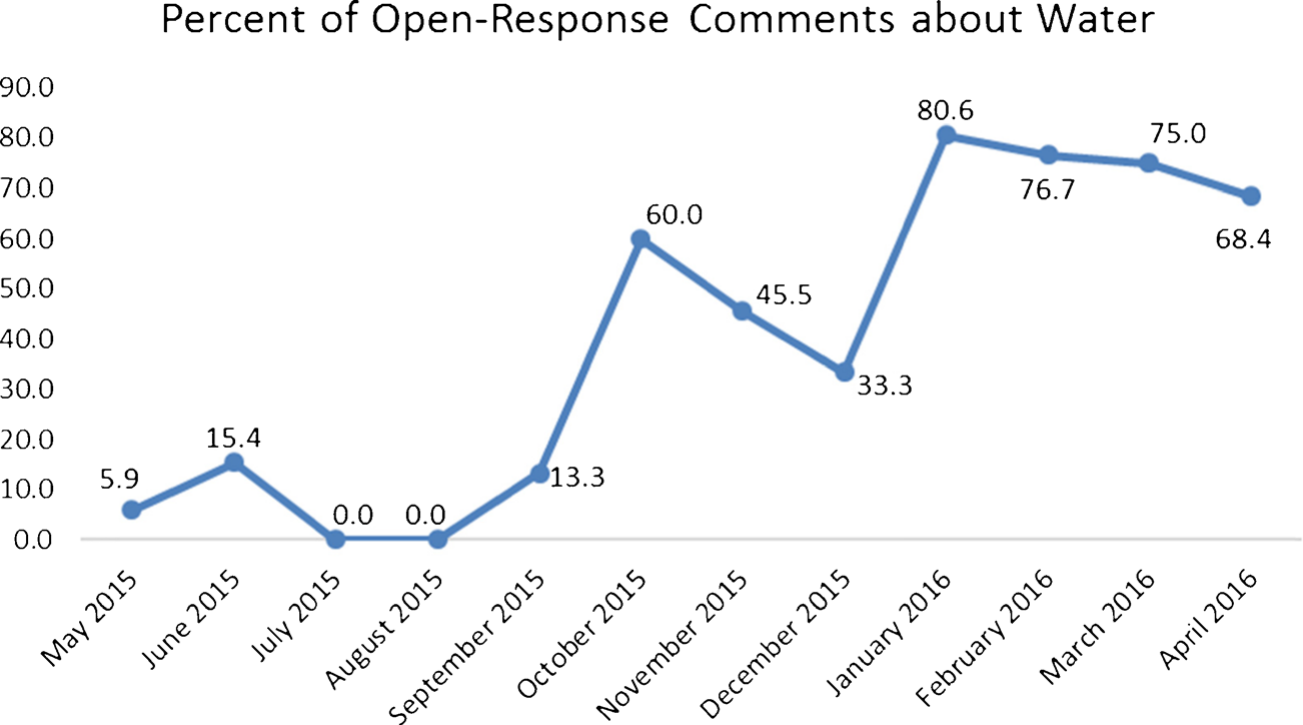
Percentage of Genesee County participants’ comments about water.

Of the 263 completed open-response questions over 12 monthly surveys, 120 were about the contamination of water in Flint (45.6 %). The number of responses to the open-response questions and the number specifically about water varied over the course of the 12 surveys but notably increased starting at survey 6 which was administered in October 2015 after Michigan state officials first publicly acknowledged that there was a problem with the water in Flint (see Fig. 2). Four comments referred to the water problems either as disastrous or a disaster, and 58 comments referred to the situation as a crisis. Quotes included from the surveys are followed by the survey date in which those responses were given.

Through the application of grounded theory coding and analysis techniques, the mental health concerns arising most clearly from the data are that the water situation has created and increased stress and anxiety for residents of Flint. From the data, stress is defined as feeling nervous, scared, angry, frustrated, and distrustful, with a lack of confidence in the political system or government. “Economic and water situation in Flint, Michigan, continues to be a focus of concern and cause of stress. Majority of families we work with reside inside the city limits and have dealt with lead and other pollutants for over a year” (January 2016). In the same survey, another panelist commented “[t]he water crisis in Flint is HUGE! Many people are very angry, scared, and unsure of the future.” In March 2016, one panelist described the situation as an emergency, leading to “toxic stress in the community.”

Stress was created by the potential and permanent physical health effects of lead exposure, the news coverage finding high levels of lead in the blood of Flint children, and lack of knowledge of where to find lead testing for children. “Parents are under more stress in seeking evaluations to identify impact of water-based lead poisoning of their children,” one panelist commented (November 2015). As another panelist frankly stated, “[r]esidents are not able to drink the Flint water. This is causing stress, anxiety, financial hardship, and community unrest.” (October 2015). Stress was perceived to increase not only because of the potential physical health effects of lead exposure but also because the solution and course of action were and remain unknown; additionally, panelists noted that residents faced extremely high water bills for unusable water.

Panelists reported that the water issues affected the whole community but would have the greatest impact among African Americans and those of low socioeconomic status in the community. These two groups were identified as those who were disproportionately receiving contaminated water and those who had the least resources to cope with it. One panelist wrote, “[r]esidents of the city of Flint are paying huge water bills and they haven’t been able to use their water in some time. It is causing a financial hardship on people that are already stretched to the max…All residents of the city of Flint are going through something because of the water issues, but it is having a devastating effect on lower income people” (October 2015). The effects of stress were not limited to Flint residents and were perceived to have a contagion effect: “The current Flint water crisis has people behaving differently due to the elevated stress level associated with living with poisoned water. Stress is through the roof for many that I come in contact with… Even those who are not directly affected are feeling stressed because they know people who are affected by it” (April 2016).

Relatedly, panelists identified anxiety and depression as increasing due to the Flint water issues for similar reasons. Panelists expressed that anxiety was elevated among community members because of the uncertainty of knowing whether they had been exposed to lead, and that the effects and severity of lead exposure would be unknown for some time.

Depression was identified as an additional mental health effect of the water problems in Flint, although it was cited much less frequently than anxiety or stress. “The water issue in our community is like…a natural disaster. It is very stressful and depressing to know that you have to pay for a commodity that you need but cannot use appropriately without potential harm to yourself and family… This increases the level of both stress and depression as it does not appear that any positive resolution is in sight” (June 2015). “I believe it [the water crisis] has exacerbated depression and some PTSD patients” (April 2016).

The mental health effects of the water issues in Flint are perceived to have spillover or ripple effects into other areas of behavioral health, such as abuse and substance use. One panelist wrote, “[t]he higher level of anxiety and stress is impacting abuse” (February 2016). Other panelists felt the water issues were causing increases in alcohol abuse, illicit drug use, and prescription misuse or abuse. “There are signs on the bars that read ‘Drink beer not the water’. In addition, people are self-medicating to deal with the mental health issues experienced” (February 2016).

Concerns about the water in Flint are indirectly affecting mental health through the effects of the situation on people living, working, and interacting in the city and county. “The Flint water situation and the fact that it’s now become a topic of national concern. It was hard living here with all the negative reporting about crime and the economy. It’s even harder living here now… People look and sound defeated,” one panelist wrote (January 2016). This individual felt that the Flint water issues were the cause of depression, stress, and hopelessness among city residents. Another panelist noted, “[t]he way people are expected to live is intolerable and weighing very heavy on people” (February 2016). “The residents seem to be on edge,” one panelist wrote (February 2016).

Beyond the perception of Flint residents experiencing anxiety and being “on edge,” there was a sense that there were mental health repercussions related to how people from other communities have begun to treat Flint residents. “There seems to be an ever increasing divide among Genesee County’s communities as everyone tries to distance themselves from Flint. You can see the ‘poor you’ look on the faces of people who find out that you live in Flint” (March 2016). Another panelist commented:

“Because of the continuing water problem in Flint, we are seeing increases in stress, substance use, and depression. Flint home property values, already down, are now in crisis mode. Some additional examples: Flint restaurants have lost business and have laid off workers, biological parents of foster children are demanding children not be placed in Flint foster homes, summer job programs for low-income county youth are being affected as families are asking for opportunities out of the city” (March 2016).

In the same month, another panelist shared that there was “increased anger from Flint homeowner residents due to [the] water crisis have identified stressful conversations due to home damage, decreased property values, illness, lack of funding to relocate, and concerns of affected family members.”

Several comments indicated a relationship between lack of confidence in government, inability to trust authority figures, and mental health. On the Flint water issues, a panelist stated, “[i]t has added stress to an already vulnerable population and created even more distrust among city and state leadership” (November 2015). Even in December 2015, after the water had been switched back to DWSD, one panelist responded, “[p]eople are unsure if the water is truly safe to drink now.” In January 2016, a panelist commented that “[t]he mayor of Flint has declared a state of emergency due to lead and chemicals in the water going into homes throughout the city. There has been an increase in anxiety and stress related to trust issues with government officials, families not being able to use or drink the water, and being required to pay a water bill for ‘bad’ water” (January 2016). In the February 2016 survey, one panelist said, “[s]ince gaining national and worldwide attention in Flint due to water crisis, we are seeing an increase in stress factors and other problems in individuals, especially children and youth. Many are interpreting the increased attention as an indicator that something much worse is unfolding that they have no power over. They state they have lost trust in believing political officials and community leaders and other than the band-aid ‘bottled water’ they are left with the consequences.” This account illustrates that mental health effects are related to distrust of the officials who should be providing solutions to the water issues, and that quick solutions are interpreted as inadequate. “Lead in the water has caused not only physical issues but being misled by city and state officials about the severity of the problem has caused stress, emotional, and mental issues, quality of life is down” (March 2016).

One panelist commented that it was not only a lack of confidence in city and government officials but a sense of abandonment that impacted mental health. One panelist responded, “how do you describe the feeling(s) of abandonment by those who you have put some degree of confidence in to protect you. In addition to now, as a result, of the water crisis, being prey to the vultures who come to feed off of the pain of this issue and all of the repercussions resulting…[this affects] predominately Flint residents, who are educated and understand the broader perspective of the impact of all of this…All of us are affected and it is unfortunate that the voice of all, the entire community is not heard” (March 2016). Part of the sense of abandonment is related to taking responsibility for the water problems in Flint: “The overall water crisis, along with the poor attempts of those who created this man-made disaster to avoid accepting responsibility… [have increased] stress, depression, anger and the list goes on!” (March 2016). Another panelist explained, “[m]ore information about the Flint water crisis is coming out as they are having legislative investigative hearings… Trust issues have led to more anxiety and depression. Substances are often used to self-medicate” (March 2016).

## Discussion

Genesee County panelists were in a unique position to assess the mental health impacts of the water issues in Flint and made astute observations about elevated levels of anxiety, stress, and depression among city residents. Panelists indicated that that the water problems were the root of increased stress, anxiety, and depression in Flint. Stress, especially prolonged or chronic stress, has the potential to lead to severe physical health outcomes such as cardiovascular disease,^24^^,^ 25 increased blood pressure, and compromised immune systems.^26^ Stress worsens other mental health indicators, such as depressive symptoms,^27^ and in combination with low socioeconomic status may lead to greater risk of premature death.^28^ Lacking positive mental health increase the probability of mortality.^29^ Stress and depression both increase chances of major cardiac events.^24^

Both the water contamination and the sense of distrust in government officials appear to have mental health consequences for Flint residents, especially as residents have no control over what flows through water service lines and into their homes. Generally, lower sense of control is associated with greater depressive symptoms^30^; in the face of disaster, decreased sense of control has been positively associated with acute stress disorder.^31^ In addition to the direct health effects of contaminated water, the mental health effects may also contribute to serious physical health reduction. Without intervention to reduce impacts, the stress effects of the water crisis and its aftermath may increase long-term health disparities faced by Flint’s predominantly poor and African American residents.

Since 2002, the City of Flint has experienced numerous fiscal challenges, leading to two explicit interventions by state government since the early 2000s. An emergency financial manager was appointed to manage Flint’s fiscal affairs from 2002 to 2004, and then again from 2012 to April of 2015.^32^ While the purpose of the intervention was to help restore the fiscal health and support the provision of basic public services, the decisions of the most recent emergency financial manager were central to the emergence and continuation of the water crisis. It could be argued that the lack of democratic checks normally present in a functioning local government hindered action that might have resulted in response to citizens’ expressed concerns about the water quality problems. In this sense, the state economic intervention appears to have resulted increased budgetary costs as well as higher human physical and mental health tolls. Michigan Governor Rick Snyder agreed with Congress in recent legislative hearings that it would be a “fair conclusion” to say that Michigan’s emergency management system failed in this case;^33^ this failure has led to not only catastrophic physical health effects but potentially long-lasting and impactful negative mental health outcomes as well.

The health and human services sector came together quickly to provide both information and interventions that address stress, anxiety, depression, and trauma brought about by the water crisis. The public mental health provider, Genesee Health System, convened the Flint Community Resilience Group (CRG) on February 2, 2016. The second meeting, held on February 16, was addressed by the US Surgeon General. At the writing of this paper, the CRG has six active workgroups: (1) planning and coordination, (2) data and gap analysis, (3) psychological first aid, (4) stress management, (5) faith-based groups, and (6) vulnerable and underserved populations. These workgroups meet regularly with the overarching purpose of providing input regarding the needs within the community, assisting with planning, and facilitating service delivery. For example, Psychological First Aid, an evidence-informed approach framed by the listen, protect, and connect approach was implemented with sessions specifically targeting adults and youth. By mid-April, 11 free-of-cost trainings were held and attended by approximately 225 individuals with an additional eight trainings scheduled between mid-April and mid-July. Data collection using the Community Assessment for Public Health Emergency Response (CASPER) was scheduled for mid-May 2016. Crisis counseling sites that connect residents to mental health services were established. The faith-based workgroup developed a presentation template that can be used at awareness summits, community conversations, workshops, and small group discussions to reduce stigma and encourage parishioners to seek mental health services.

The water crisis served to draw professionals and community members together to support the Flint residents who are in need of behavioral health services. Under the leadership of the Genesee Health System, a Community Resilience Group (CRG) was formed in February 2016 to address the behavioral health issues rising from the crisis. A Community Response Team coordinated by the Red Cross is prioritizing mental health and coordinates its efforts with the CRG. The workgroups are planning and coordinating the following efforts: stress management/workforce health protection, assisting vulnerable and underserved populations, providing psychological first aid, conducting data and gap analysis, and faith-based group activities. The CRG has produced a draft Behavioral Health Recovery and Resilience Plan composed around a set of values: living-empowering individuals to thrive in the face of adversity; healing-connecting individuals to the care needed to restore health and wellness; trust-restoring confidence in community resources and agencies; hope-instilling an expectation of a better future; connectedness-fostering relationships among community partners and residents; and knowledge-ensuring timely/accurate information. The plan addresses short-term, intermediate-term, and long-term goals for each of the aforementioned workgroups.

Panelists identified several mental health issues that were increasing as the water issues in Flint unfolded. Like the physical health consequences of lead poisoning, the full extent of mental health consequences may not be known for some time. Efforts to provide funding or services for the health consequences of lead and other contaminant exposure would be remiss if they did not include coverage for mental health effects many residents continue to face. Indeed, mental health services should be incorporated as part of the emergency response^34^ and carried forward for the next several years.^35^
